# Sex hormones and brain volumes in a longitudinal study of middle‐aged men in the CARDIA study

**DOI:** 10.1002/brb3.765

**Published:** 2017-09-20

**Authors:** Martine Elbejjani, Pamela J. Schreiner, David S. Siscovick, Stephen Sidney, Cora E. Lewis, Nick R. Bryan, Lenore J. Launer

**Affiliations:** ^1^ Laboratory of Epidemiology and Population Science National Institute on Aging Bethesda MD USA; ^2^ Division of Epidemiology and Community Health University of Minnesota Minneapolis MN USA; ^3^ School of Public Health University of Washington Seattle WA USA; ^4^ Division of Research Kaiser Permanente Northern California Oakland CA USA; ^5^ Division of Preventive Medicine University of Alabama at Birmingham Birmingham, AL USA; ^6^ Department of Radiology University of Pennsylvania Health System Philadelphia PA USA; ^7^ The New York Academy of Medicine New York, NY USA

**Keywords:** brain volumes, men's health, MRI, sex hormone binding globulin, testosterone

## Abstract

**Introduction:**

Several findings suggest that testosterone (T) is neuroprotective and that declining T levels during aging are associated with cognitive and brain pathologies; however, little is known on T and brain health in middle‐age. We examined the relationships of total T, bioavailable T, and sex hormone binding globulin (SHBG) levels with total and regional gray matter (GM) and white matter (WM) volumes in middle‐aged men. We also evaluated the association of sex hormone levels with cognitive function.

**Methods:**

Analysis included 267 community‐dwelling men participating in the Coronary Artery Risk Development in Young Adults (CARDIA) brain magnetic resonance imaging (MRI) substudy. Total T, bioavailable T, and SHBG levels were measured at three times from the 2^nd^ to 4^th^ decade of life; brain volumes were measured at the ages of 42–56. Associations were estimated using linear regression models, adjusted for several potential confounders.

**Results:**

Higher SHBG levels were associated with greater total WM volume (+3.15 cm^3^ [95% confidence interval [CI] = 0.01, 6.28] per one standard deviation higher SHBG). Higher SHBG levels were associated with lower total and regional GM volumes overall and significantly with smaller parietal GM volume (−0.96 cm^3^ [95%CI = −1.71, −0.21]). T levels were not related to brain volumes. Neither T nor SHBG levels were associated with cognitive function.

**Conclusion:**

Results suggest a role for SHBG in structural brain outcomes in men and emphasize the value of investigating SHBG levels as modulators of sex hormone and metabolic pathways regulating brain and behavioral characteristics in men.

## 1 INTRODUCTION

Prior research suggests that the male sex hormone testosterone (T) influences brain structures and functions (Cahill, 2006). Measures of T include total‐T and bioavailable T (bio‐T), the active portion (40%–65%) of total‐T that is not bound to sex hormone globulin (SHBG) and available to interact with androgen receptors (Ronde et al., 2005). SHBG is a major binding protein for T and a key regulator of bio‐T (Holland, Bandelow, & Hogervorst, 2011; Ronde et al., 2005). Experimental studies show that T has neuroprotective properties and report positive links between T levels and several brain processes related to cognitive function, such as increased neuroplasticity, neurogenesis, β‐amyloid regulation, and larger gray matter (GM) volume (Fanaei et al., 2014; Galea, 2008; Gouras et al., 2000; Holland et al., 2011). Similarly, studies in boys report that T levels during childhood and adolescence are related to brain development and organization (Blakemore, Burnett, & Dahl, 2010; Herting, Gautam, Spielberg, Dahl, & Sowell, 2015). Studies in men have focused predominantly on older age. It is well‐established that T levels decrease with age; and, this aging‐driven decline in total‐T or bio‐T levels has been associated with increased risk of dementia, cognitive decline (Holland et al., 2011; Moffat et al., 2002; Verdile et al., 2014; Yaffe, Lui, Zmuda, & Cauley, 2002), and dementia‐related neuropathology (Strozyk et al., 2007; Verdile et al., 2014). Increasing SHBG levels during aging have also been linked to cognitive disorders (Caldwell & Jirikowski, 2009; Muller, Schupf, Manly, Mayeux, & Luchsinger, 2010).

Few data exist on sex hormones and brain measures in young adult to middle‐aged men. Given the wide interest in using T supplements for several aging‐related conditions, including cognitive impairment, research in this younger age‐period can inform on how earlier relationships of T and brain structures may contribute to aging‐related effects of T on brain and cognitive changes.

We examined the relationships of total‐T, bio‐T, and SHBG levels in young adulthood (2^nd^ to 4^th^ decade of life) and brain volumes at middle‐age (4^th^–5^th^ decade) in a prospective cohort of community‐dwelling men. Previous research suggests that sex hormones may present differential patterns of associations with distinct brain regions — including ones that are related to dementia – and with specific brain functions (Holland et al., 2011; Lessov‐Schlaggar et al., 2005; Peper & Koolschijn, 2012; Witte, Savli, Holik, Kasper, & Lanzenberger, 2010). We therefore examined both global and regional GM and white matter (WM) volumes to identify brain regions that may be differently related to T and SHBG levels in middle‐age men; in secondary analyses, we assessed the relationship of T and SHBG levels with three components of cognitive performance: psychomotor speed, verbal memory, and executive function.

## 2 MATERIALS AND METHODS

### 2.1 Study population

**Figure 1:**
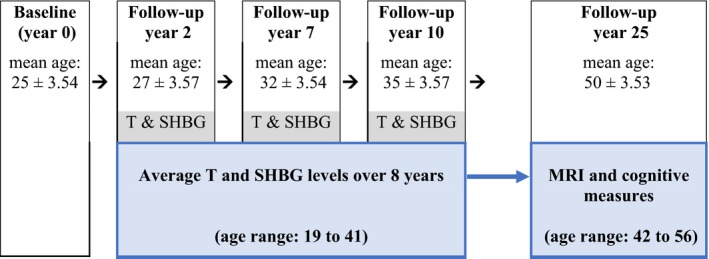
Timeline of sex hormone and brain measures in the male participants in the Coronary Artery Risk Development in Young Adults study. *N* = 267 with complete MRI and sex hormone data; *n* = 262 with complete sex hormone and cognitive data. T, testosterone; SHBG, sex hormone binding globulin; MRI, magnetic resonance imaging

The Coronary Artery Risk Development in Young Adults (CARDIA) study is a multi‐center population‐based longitudinal study of the determinants of cardiovascular disease in young black and white adults (Friedman et al., 1988). At baseline (1985–1986), 5,115 participants (2,328 men) aged 18–30 years were enrolled in four US cities (Birmingham, Alabama; Chicago, Illinois; Minneapolis, Minnesota; and Oakland, California). Participants were followed for 25 years (with examinations at years 2, 5, 7, 10, 15, 20, and 25 of follow‐up); 72% (*n* = 3,498) of the total baseline cohort (65% of the male subjects (*n* = 1,517)) participated in the 25^th^ year examination. We used data from two CARDIA subsamples: (1) the CARDIA Male Hormone Study (CMHS), (*n* = 1420), which collected repeated measurements of total‐T, bio‐T, and SHBG serum concentrations at the 2^nd^, 7^th^, and 10^th^ year examinations (at mean ages 27.05 [±3.57], 32.05 [±3.54], and 35.02 [±3.57], respectively) (Gapstur et al., 2002); and (2) the CARDIA Brain MRI subsample, which included participants in Birmingham, Minneapolis, and Oakland who were invited to participate in a brain MRI examination (*n* = 719) at the year 25 visit (at the ages of 42–56); the design and timeline of this investigation are presented in Figure 1. There were 342 male participants in the MRI examination; of which 334 (98%) had good quality scans, and of those, 267 had also participated in the hormone study. The 267 men with MRI and hormonal data constituted our analytical sample.

Each participant provided written informed consent at each exam and the study was annually approved by the institutional review boards at each site (Launer et al., 2015).

### 2.2 Data

#### 2.2.1 MRI measures

Brain MRIs were performed using 3‐T MR scanners (Oakland: Siemens 3T Tim Trio/VB 15 platform; Minneapolis: Siemens 3T Tim Trio/VB 15 platform; and Birmingham: Philips 3T Achieva/2.6.3.6 platform). At each center, scans were performed according to standardized protocols following the supervision of the MRI Reading Center (University of Pennsylvania) (Launer et al., 2015). Image processing, quality control checks, and automated brain tissue volume computations were performed at the MRI reading center, following previously described protocols (Goldszal et al., 1998; Lao et al., 2008; Launer et al., 2015; Shen & Davatzikos, 2002; Zacharaki, Kanterakis, Bryan, & Davatzikos, 2008). In brief, all imaging data were first converted to Neuroimaging Informatics Technology Initiative (NIFTI) format. Initial quality control (QC) was performed through manual inspection of each subject's T1 scan for motion artifacts and other quality issues. The preprocessing of the T1‐weighted scan involved brain extraction (i.e., the removal of the skull, extracerebral tissues, and cerebellum) using a multiatlas segmentation method (Doshi, Erus, Ou, Gaonkar, & Davatzikos, 2013), correction of image inhomogeneities (Sled, Zijdenbos, & Evans, 1998), and segmentation of the brain parenchyma into GM, WM and cerebrospinal fluid (Li, Gore, & Davatzikos, 2014). Total brain volume was obtained by summing GM and WM volumes; total intracranial volume was the sum of GM, WM, and cerebral spinal fluid volumes. The brain was segmented into anatomical regions by transferring expert defined region of interest (ROI) labels on a standard template image space to the subject T1 space through nonlinear registration (Shen & Davatzikos, 2002). GM and WM were classified into regions of interest according to the Jakob atlas (Kabani, Collins, & Evans, 1998) and further into normal and abnormal and tissue. Within each ROI, GM and WM volumetric measurements are calculated. Abnormal WM included damaged tissue resulting from ischemia, demyelination, inflammation, and damaged penumbra tissue surrounding focal infarcts; the amount of abnormal GM and WM was, on average, very low and hence we did not report these data separately.

##### Outcome measures

We investigated (1) global brain volume measures: total brain, GM, and WM volumes and (2) lobar GM and WM tissue volumes. In additional analyses, we assessed whether sex hormone levels were associated with volumes of certain brain structures linked to cognitive disorders: the prefrontal cortex, medial‐temporal cortex, hippocampus, amygdala, and the entorhinal cortex (Brand & Markowitsch, 2008; Desikan et al., 2009).

#### 2.2.2 Hormone data

Blood samples were collected by venipuncture between 07:30 and 12:00 hr and aliquots of serum were stored at −70°C. Total‐T was measured using radioimmunoassay; SHBG was measured by chemiluminescent enzyme immunometric assay (Immulite, Diagnostic Products Corporation, Los Angeles, CA, USA) (Gapstur et al., 2002). Assay variability was monitored by including 10% blind quality control samples in each batch of samples analyzed. Intra and interbatch variations were 12.3% and 11.2% for total‐T, and 7.9% and 11.2% for SHBG. There was no association between time of blood draw and hormone levels (Gapstur et al., 2002). Bio‐T levels were calculated based on the Sodergard et al., method (Södergard, Bäckström, Shanbhag, & Carstensen, 1982; Vermeulen, Verdonck, & Kaufman, 1999).

Of the 267 men, 233 had three valid sex hormone measurements, 31 had two valid measures and three men had only one hormone measurements at Year 10. Given that T and SHBG levels in men do not typically begin to change significantly before the late third or early fourth decade of life (Allan & McLachlan, 2004) and given that, in our sample, repeated measurements of each of T and SHBG levels showed moderate‐to‐strong correlations per individual (Pearson correlation between repeated values of each sex hormone of interest ranging from 0.5 to 0.8), we averaged the repeat measures (ng/ml) together to obtain a stable estimate of exposure to the hormones over the 8‐year interval between exams 2 and 10; for the 3 men with only one measurement of sex hormones, we used their Year 10 value. We converted the average values to z‐scores to facilitate interpretability across the different hormone levels investigated. In sensitivity analyses, we evaluated the association of brain volumes to the individual sex hormone levels at the three time‐points as well as the change in sex hormone levels from Y2 to Y10.

#### 2.2.3 Covariates

Based on their associations with brain volumes and/or hormonal measures (Bain, 2010; Caldwell & Jirikowski, 2009; Friedman et al., 2014) in the literature and/or in our sample, several covariates were included in the analyses: age, race (black/white), educational attainment (≤high school, >high school), total intracranial volume, body mass index, smoking status (never‐smoker, ex‐smoker, current smoker), hypercholesterolemia (total cholesterol ≥ 240 mg/dl and/or use of cholesterol lowering medication), fasting insulin measures (uU/ml), APOE‐e4 genotype (e4‐allele carriers, noncarriers) and medical history of: depressive symptoms (current or prior Center for Epidemiologic Studies‐Depression CES‐D score ≥ 16 and/or use of medication for depression), hypertension (current or prior diastolic blood pressure ≥ 90 and/or systolic blood pressure ≥ 140 and/or taking antihypertensive medication), vascular disorders (self‐reported occurrence of: heart attack, angina, heart failure, rheumatic heart disease, mitral valve prolapse, stroke and peripheral vascular disease), and diabetes mellitus status (current or prior diabetes defined following ADA criteria for levels of fasting, nonfasting or postprandial oral glucose tolerance test (OGTT) results, Hemoglobin A1c (HbA1c) percent, or use of antidiabetes medication). We used the Year 25 values of these covariates as most represented their history/cumulative values which potentially occurred in parallel to the sex hormone exposures of interest.

### 2.3 Statistical analysis

Multivariable linear regression models were used to estimate the separate associations of each of total‐T, bio‐T, and SHBG levels (entered individually in linear regression models) with each brain volume of interest (global, lobar, and candidate structures’ volumes). We ran two sets of models: Model 1 adjusted for age, race, educational attainment, and total intracranial volume; Model 2 adjusted for all potential confounders described above and for the study centers. In secondary analyses, we estimated the associations of sex hormone levels with cognitive function assessed at Year 25 using the Digit Symbol Substitution Test (DSST, psychomotor speed), the Rey Auditory Verbal Learning Test (RAVLT, verbal memory), the Stroop interference test (executive function), and a composite cognitive score (combining the z‐scores of the three tests).

Linearity of the associations between sex hormone levels and brain volumes was accessed with fractional polynomial models (Royston, Ambler, & Sauerbrei, 1999). All statistical analyses were performed using SAS software version 9.3 (SAS Institute, Cary NC 2011, RRID: SCR_008567).

## 3 RESULTS

Mean age at time of the MRI was 50 years (±3.5 years); total‐T, bio‐T, and SHBG levels (ng/ml), averaged over 8 years, were normally distributed around means of 6 ± 1.6, 3 ± .8, and 30 ± 10, respectively (Table 1). Global and regional GM and WM volumes were also normally distributed.

**Table 1:** CARDIA men subsample with hormone and neuroimaging data

*n* = 267	Mean or *n*	SD or %	*N* missing
Age	50	3.53	0
Race (white)	173	64.79%	0
Education (high school or less)	62	23.22%	0
Smoking status			1
Never‐smoker	170	63.91%	
Ex‐smoker	55	20.68%	
Smoker	41	15.41%	
Apolipoprotein E‐e4 genotype (e4 carrier)	79	30.98%	12
History of diabetes mellitus status	27	10.11%	0
History of cardiovascular events	30	11.24%	0
History of hypertension	103	38.58%	0
History of depressive symptoms	102	38.20%	0
Body mass index	28.68	4.57	0
Fasting insulin (uU/ml)	11.18	9.48	0
Hypercholesterolemia	59	22.10%	0
Cognitive composite score	−0.20	2.28	5
*Sex hormone measures*			
Measures at each time‐point			
Year 2 follow‐up			
Total‐T (ng/ml)	6.34	1.69	11
Bio‐T (ng/ml)	3.15	0.94	11
SHBG (ng/ml)	31.35	10.44	11
Year 7 follow‐up			
Total‐T (ng/ml)	5.87	1.98	26
Bio‐T (ng/ml)	2.93	1.09	26
SHBG (ng/ml)	29.90	11.80	26
Year 10 follow‐up			
Total‐T (ng/ml)	5.70	1.75	0
Bio‐T (ng/ml)	2.80	0.92	0
SHBG (ng/ml)	29.25	11.56	0
Mean levels over 8 years^a^			
Total‐T (ng/ml)	6.01	1.60	0
Bio‐T (ng/ml)	2.97	0.82	0
SHBG (ng/ml)	30.06	10.28	0
*Cerebral volumes*			
Global volumes (cm^3^)			
Total brain	1048.69	101.14	0
Total GM	548.48	52.21	0
Total WM	500.21	56.22	0
Lobar GM volumes (cm^3^)			
Frontal GM	172.12	13.39	0
Parietal GM	99.99	11.33	0
Temporal GM	139.88	13.98	0
Occipital GM	67.81	8.37	0
Lobar WM volumes (cm^3^)			
Frontal WM	201.72	24.65	0
Parietal WM	92.53	10.40	0
Temporal WM	119.04	13.55	0
Occipital WM	49.41	7.43	0

T, testosterone; bio‐T, bioavailable testosterone; SHBG, sex hormone binding globulin; CARDIA, Coronary Artery Risk Development in Young Adults; GM, gray matter; WM, white matter.

a Mean of the year 2, 7, and 10 measurements.

Total‐T levels were positively correlated with bio‐T and SHBG levels (Pearson's correlation = .66 [*p *<* *.0001] and .54 [*p* < .0001], respectively); bio‐T and SHBG concentrations were weakly negatively correlated (correlation = −.11 [*p* = .07]).

Total brain volume was not related to sex hormone concentrations (Model 1 coefficients for T = −1.33 [95% CI = −5.08, 2.41], bio‐T = −1.23 [95% CI = −5.03, 2.57], and for SHBG = 0.40 [95% CI = −3.33, 4.13]).

There was an overall pattern of associations, although not significant, of both higher total‐T and lower SHBG levels with smaller total GM volume (Model 1; Table 2). Adjusting for covariates (Model 2; Table 2), higher SHBG levels were associated with significantly smaller parietal GM volume (with a 0.96 cm^3^ [95% CI = −1.71, −0.21; *p* = .012] smaller parietal volume per one z‐score [corresponding to a 10.28 ng/ml] increase in SHBG level); further adjustment for total‐T levels did not change this association (coefficient = −1.14; 95% CI = −2.01, −0.26; *p* = .01). Higher bio‐T levels were associated with larger parietal GM volume but this association was not statistically significant after adjusting for relevant covariates (Table 2; Model 2) and separately for SHBG levels (coefficient = 0.48; 95% CI = −0.31, 1.27; *p* = .23). Higher total‐T and SHBG levels were associated with lower occipital GM in Model 1, but the association with total‐T was attenuated after adjustment for covariates.

**Table 2:** Associations of hormone levels with total and lobar gray matter (GM) brain volumes: CARDIA men subsample with hormone and neuroimaging data

	GM volumes							
	Model 1^a^				Model 2^b^			
	Beta	95% CI	*p*	Adjusted *R* ^2^	Beta	95% CI	*p*	Adjusted *R* ^2^
*Total GM*								
Total‐T	−2.53	−5.32, 0.26	.075	.810	−2.22	−5.27, 0.82	.151	.820
Bio‐T	0.21	−2.64, 3.06	.887	.807	0.31	−2.79, 3.42	.843	.818
SHBG	−2.75	−5.52, 0.02	.052	.810	−2.08	−5.03, 0.87	.166	.820
*Frontal GM*								
Total‐T	−0.90	−2.04, 0.28	.125	.741	−0.80	−2.08, 0.48	.219	.743
Bio‐T	−0.09	−1.26, 1.08	.881	.739	−0.05	−1.36, 1.26	.939	.741
SHBG	−0.65	−1.79, 0.49	.263	.740	−0.42	−1.66, 0.83	.506	.742
*Temporal GM*								
Total‐T	−0.51	−1.44, 0.43	.286	.702	−0.21	−1.24, 0.81	.685	.716
Bio‐T	0.003	−0.95, 0.96	.994	.700	0.06	−0.98, 1.11	.908	.716
SHBG	−0.59	−1.52, 0.34	.209	.702	−0.19	−1.19, 0.79	.692	.716
*Parietal GM*								
Total‐T	−0.12	−0.82, 0.58	.733	.701	−0.35	−1.13, 0.43	.386	.702
Bio‐T	0.82	0.12, 1.52	.023	.707	0.64	−0.15, 1.42	.117	.704
SHBG	−0.86	−1. 45, −0.18	.014	.708	−0.96	−1.71, −0.21	.012	.709
*Occipital GM*								
Total‐T	−0.71	−1.46, 0.04	.062	.471	−0.61	−1.35, 0.15	.115	.569
Bio‐T	−0.26	−1.02, 0.50	.506	.464	−0.10	−0.86, 0.67	.805	.564
SHBG	−0.70	−1.44, 0.05	.065	.470	−0.63	−1.35, 0.09	.087	.570

Levels of total‐T, bio‐T, and SHBG expressed in z‐scores; estimated beta coefficients are the differences in brain volumes per one standard deviation increase in predictor levels (which correspond to a value of 1.6, 0.82, and 10.28 [ng/ml] for total‐T, bio‐T, and SHBG, respectively).

CI, confidence interval; T, testosterone; Bio‐T, bioavailable testosterone; SHBG, sex hormone binding globulin; CARDIA, Coronary Artery Risk Development in Young Adults.

a Model 1 adjusted for age, education, race, and total intracranial volume.

b Model 2 adjusted for age, education, race, total intracranial volume, study center, body mass index, fasting insulin, hypercholesterolemia, smoking status, Apolipoprotein E e4 genotype, and history of vascular and cerebrovascular conditions, diabetes mellitus status, hypertension, and depressive symptoms.

Higher SHBG levels were associated with significantly greater total WM volume (with a 3.15 cm^3^ [95%CI = 0.01, 6.28] greater WM volume per one z‐score increase in SHBG level; Table 3); this association remained unchanged after adjusting for all covariates (Model 2). SHBG levels were not significantly associated with WM volumes in specific lobes; however, associations were more pronounced with temporal and frontal WM volumes (coefficient = 0.91 [*p* = .06] and coefficient = 1.59 [*p* = .07], respectively, in fully adjusted Model 2).

**Table 3:** Associations of hormone levels with total and lobar white matter (WM) brain volumes: CARDIA men subsample with hormone and neuroimaging data

	WM volumes							
	Model 1^a^				Model 2^b^			
	Beta	95% CI	*P*	Adjusted *R* ^2^	Beta	95% CI	*p*	Adjusted *R* ^2^
*Total WM*								
Total‐T	1.20	−1.98, 4.37	.458	.788	1.31	−2.19, 4.82	.461	.787
Bio‐T	−1.43	−4.65, 1.78	.381	.788	−2.14	−5.70, 1.41	.236	.787
SHBG	3.15	0.01, 6.28	.049	.791	3.42	0.05, 6.79	.047	.790
*Frontal WM*								
Total‐T	0.45	−1.19, 2.09	.588	.704	0.73	−1.06, 2.52	.425	.710
Bio‐T	−0.77	−2.43, 0.90	.366	.705	−0.75	−2.56, 1.07	.420	.710
SHBG	1.47	−0.16, 3.09	.077	.708	1.59	−0.13, 3.32	.070	.713
*Temporal WM*								
Total‐T	0.17	−0.71, 1.04	.708	.724	0.30	−0.68, 1.27	.549	.718
Bio‐T	−0.26	−1.14, 0.63	.566	.724	−0.42	−1.41, 0.57	.405	.718
SHBG	0.69	−0.17, 1.56	.115	.726	0.91	−0.02, 1.85	.056	.722
*Parietal WM*								
Total‐T	0.31	−0.52, 1.14	.468	.710	0.18	−0.74, 1.10	.702	.710
Bio‐T	−0.19	−1.03, 0.66	.667	.710	−0.40	−1.33, 0.53	.400	.710
SHBG	0.43	−0.40, 1.26	.307	.711	0.30	−0.59, 1.19	.512	.710
*Occipital WM*								
Total‐T	0.28	−0.45, 1.01	.446	.362	0.25	−0.49, 0.99	.508	.476
Bio‐T	−0.12	−0.86, 0.62	.749	.361	−0.36	−1.11, 0.38	.337	.477
SHBG	0.34	−0.36, 1.08	.324	.363	0.51	−0.20, 1.22	.162	.479

Levels of total‐T, bio‐T, and SHBG expressed in z‐scores; estimated beta coefficients are the difference in brain volumes per one standard deviation increase in predictor levels (which correspond to a value of 1.6, 0.82, and 10.28 [ng/ml] for total‐T, bio‐T, and SHBG, respectively).

CI, confidence interval; T, testosterone; Bio‐T, bioavailable testosterone; SHBG, sex hormone binding globulin; CARDIA, Coronary Artery Risk Development in Young Adults.

a Model 1 adjusted for age, education, race, and total intracranial volume.

b Model 2 adjusted for age, education, race, total intracranial volume, study center, body mass index, fasting insulin, hypercholesterolemia, smoking status, Apolipoprotein E e4 genotype, and history of vascular and cerebrovascular conditions, diabetes mellitus status, hypertension, and depressive symptoms.

### 3.1 Additional analyses

Sex hormone levels were not associated with the GM structures, hippocampus, amygdala, entorhinal cortex, prefrontal cortex, or medial‐temporal cortex volumes (Table S1). There was no indication of nonlinearity in the associations between sex hormone levels and the brain volumes. Sex hormone concentrations were not associated with cognitive scores (Supplementary Table S2). In additional analyses, we examined changes in T and SHBG levels between year 2 and year 10. The changes were small (mean = 0.66 [SD = 1.59] for total‐T, 0.35 [SD = 0.97] for bio‐T, and 1.96 [SD = 8.44] for SHBG levels) and not associated with brain volumes (all *p* values >.10). The sex hormone measures at each time‐point showed comparable associations as those found with the averaged hormone measurements: SHBG levels across all time‐points were associated with smaller parietal GM, associations of SHBG levels with total WM were stronger with the Y7 and Y10 measurements (Table S3).

## 4 DISCUSSION

In this cohort of community‐dwelling men, SHBG levels measured over 8 years in young adulthood were associated with both global and regional brain volumes measured 15 years later, at middle‐age. Higher SHBG levels were associated with greater total WM volume with associations more pronounced in the temporal and frontal lobes. We also found a regional association of higher SHBG levels and smaller GM volume in the parietal lobe. There was an overall pattern of associations between higher total T levels and smaller total GM volume but these associations were not statistically significant. Cognitive functioning was not related to SHBG or T levels, which is consistent with several studies in older adults (Lessov‐Schlaggar et al., 2005; Moffat et al., 2002; Yaffe et al., 2002).

Overall, results suggest that SHBG levels in young adulthood may contribute to some of the variability in WM and GM volumes in middle‐aged men. There are few studies investigating prospectively the association of male sex hormone levels and brain health in adulthood. Concordant with our findings, a study of healthy older men (Lessov‐Schlaggar et al., 2005), also reported no association between T or SHBG levels, collected at the ages of 52 to 70, and total brain volume or cognitive function measured 10–16 years later (Lessov‐Schlaggar et al., 2005). Furthermore, the regional associations found in that study and ours generally overlap, although a direct comparison of the findings is limited because that study did not differentiate between GM and WM tissue volume. One major difference is that Lessov‐Schlaggar et al. (2005) observed in their older sample associations with higher T levels, but not SHBG, and larger parietal and frontal lobes’ volumes and smaller occipital lobe volumes.

Accumulated findings regarding the role of SHBG in metabolic health and aging‐related conditions (Caldwell & Jirikowski, 2009; Li et al., 2015) may shed some light on these different findings. Vascular and metabolic factors are among the most important risk factors mediating brain health and neurocognitive outcomes. Lower SHBG levels have been widely associated with a higher risk of metabolic conditions, including diabetes, cholesterolemia, and obesity, and with adverse changes in fasting insulin, glucose, and BMI (Bhasin et al., 2011; Caldwell & Jirikowski, 2009; Laaksonen et al., 2004). Furthermore, studies have linked lower SHBG levels to several aging‐related disorders that affect various biological pathways, such as dementia, vascular disorders, and osteoporosis (Caldwell & Jirikowski, 2009). Such findings indicate a close relationship between SHBG levels and various basic processes (Caldwell & Jirikowski, 2009; Laaksonen et al., 2004; Li et al., 2015) and emphasize its role as a marker of several regulatory mechanisms in the body and the brain, including levels of T and of important metabolic factors. This is in line with findings by Hogervorst, Bandelow, Combrinck, & Smith (2004), which showed that SHBG levels were associated with risk of Alzheimer's disease in younger older men (mean age = 66) but that total testosterone levels were associated with the disease in the older participants (mean age = 80) (Hogervorst et al., 2004). SHBG levels at younger age may thus be acting as an indicator of general metabolic and/or regulatory factors and potentially as an earlier marker of changes in these factors and of the subsequent effects of these changes on brain health. Our Model 2 results indicate a more direct link between SHBG levels and brain volumes that is potentially independent of metabolic factors and conditions in middle‐aged men, although we can't exclude residual confounding.

One interesting finding is that higher SHBG levels were associated with larger WM volume but smaller GM volume in our sample. It is not evident why SHBG levels would be differently related to GM and WM brain tissue. The widespread role of SHBG in regulating both metabolic and sex hormone pathways may help explain this finding, wherein SHBG might be related to WM via more metabolic pathways and to GM through sex‐hormone‐related pathways. The association between higher SHBG levels and larger WM volume may thus be reflecting the hypothesized role of SHBG as an encompassing indicator of general and metabolic health, as higher SHBG levels are typically related to better metabolic outcomes (Caldwell & Jirikowski, 2009). Consistently, higher SHBG levels were associated with lower BMI and insulin levels in our sample (Table S4). WM alterations, such as compromised microstructural integrity, volume loss, and WM lesions have been linked to aging (Gunning‐Dixon, Brickman, Cheng, & Alexopoulos, 2009) and poorer cardio‐metabolic health (Launer et al., 2015; Segura et al., 2009). In parallel, the association of higher SHBG levels with smaller GM volume may be related to SHBG's role in regulating sex‐hormone‐related mechanisms, wherein higher SHBG typically indicate lower bioavailable T and estrogen levels. This is consistent with our results of a trend of association of total T with GM volume and of an association between higher bio‐T levels and smaller parietal GM volume. The latter association was not significant after adjusting for relevant covariates or SHBG levels. This suggests that the positive association between bio‐T levels and parietal GM could be reflecting the association of lower SHBG levels and larger parietal GM volume, and hence when models adjusted directly (including SHBG in the model) or indirectly for confounding by SHBG (via adjustment for factors related to SHBG, such as insulin and BMI‐ Table S4), the results with Bio‐T were attenuated.

It is not clear why SHBG levels would only be significantly related to the parietal GM. Other studies have previously reported gender‐differences in the structure and development of the parietal lobe (Peper, Hulshoff Pol, Crone, & van Honk, 2011; Salinas et al., 2012). However, volumes of other regions in the brain also show sex differences. Some imaging studies have reported age‐differences in the function of the parietal cortex and an increased sensitivity of the parietal lobe to the aging‐driven brain atrophy (Jacobs et al., 2011; Resnick, Pham, Kraut, Zonderman, & Davatzikos, 2003). Thus, parietal GM may be a brain region where the involvement of SHBG in both sex hormone and metabolic pathways converge in men. Overall, our results strengthen the rationale of examining the role of SHBG at different ages and the different mechanisms by which it relates to brain structure and function in men and whether similar associations can be found in women.

### 4.1 Strengths and limitations

Strengths of our study include the community‐based sample, using repeated measurements of sex hormone levels (which could have minimized the impact of measurement error and represented a cumulative/longitudinal exposure to sex hormone concentrations and which allowed to evaluate hormone levels at different ages and change in hormone levels), the investigations of global and regional brain volumes, and adjustment for several potential confounders. Certain associations increased in magnitude when covariates were included; this could be explained by the presence of important associations of certain covariates with hormone levels (BMI, insulin level) and with brain measures (hypertension, diabetes, and smoking status) (Tables S4 and S5), which could have thus reduced the variability in hormone and brain measures. This is line with other observations of the importance of these covariates in studies of T and neurocognitive pathology in older cohorts (Holland et al., 2011). One limitation is the small sample size which could have hindered the detection of certain associations. Our study is the first investigation of the relationship between T and brain volumes in middle‐age men and included examinations of several components for T levels (total‐T, bio‐T, and SHBG) as well as global and regional volumes; the study thus tested multiple hypotheses which inflate type I error rate and associations will not be significant following a Bonferroni correction for multiple testing. Results should be interpreted in the light of this limitation. Nonetheless, results present consistent patterns suggesting that they may not be driven by chance; specifically, the consistency of the association of SHBG with total WM volume and then with the same regional WM (in the frontal and temporal lobes) and with parietal GM across different time‐points (Table S3). Hormonal measures were collected 10–15 years prior to the brain measures and our results could reflect a temporal association of sex hormones in early adulthood and brain volumes in mid‐life. However, it is also possible that unmeasured factors, such as earlier brain characteristics and/or common pathways (i.e., developmental, environmental, and/or genetic mechanisms), might have influenced both hormonal profiles and brain volumes in early adulthood and mid‐life and resulted in their observed associations. Given the involvement of sex hormone levels in several developmental and biological processes and given data indicating that they are often involved in bidirectional mechanisms (Allan & McLachlan, 2010; Kim & Halter, 2014), it is possible that their association with brain health is also bidirectional. Future studies are needed to establish the temporality of the association throughout the life‐course.

Our study lacked information on estrogen concentrations, which are closely related to T and SHBG levels and which have been linked to brain volumes (Strozyk et al., 2007). Bio‐T levels were not measured directly (i.e., by equilibrium dialysis), which might have resulted in measurement error. However, this error is not likely to be systematically related to the brain outcomes. Also, several formula‐based bio‐T estimations, including the one used in CARDIA, were found to provide valid and reliable measurements of bio‐T (Ly et al., 2010).

## 5 CONCLUSION

In this first investigation of the links between cumulative sex hormone levels in young adulthood and brain volumes measured at middle‐age in men, higher SHBG levels were associated with larger total WM volume. Regionally, this association was more pronounced with WM volumes in the frontal and temporal lobes. Higher SHBG levels were linked to significantly smaller parietal GM. Results emphasize the value of including SHBG in studies of sex hormones and brain outcomes and suggest that in middle‐age men, SHBG levels—which might reflect differential regulation of sex hormones and aging‐related changes—are related to GM and WM volumes.

## CONFLICT OF INTEREST

All authors report no disclosures.
