# Supplementary material for: Sex hormones and brain volumes in a longitudinal study of middle‐aged men in the CARDIA study
Source: Brain Behav. 2017 Sep 20;7(10):e00765. doi: 10.1002/brb3.765 (PMC5651379; doi:10.1002/brb3.765)
Supplement: Supplementary file 1 [file BRB3-7-e00765-s001.docx]

**SUPPLEMENTARY TABLES**

**Supplementary Table 1. Sex-hormones levels and brain tissue volumes of regions associated with cognitive functioning: CARDIA men sub-sample with hormone and neuroimaging data**

|  | **Model 1^a^** | | **Model 2^b^** | |
| --- | --- | --- | --- | --- |
|  | Beta | 95% CI | Beta | 95% CI |
|  | **Hippocampal volume** | | | |
| **Total-T** | -0.01 | -0.10, 0.08 | 0.03 | -0.07, 0.13 |
| **Bio-T** | 0.01 | -0.08, 0.14 | 0.04 | -0.06, 0.14 |
| **SHBG** | -0.04 | -0.13, 0.05 | -0.02 | -0.11, 0.08 |
|  | **Amygdala volume** | | | |
| **Total-T** | -0.01 | -0.05, 0.03 | -0.01 | -0.05, 0.04 |
| **Bio-T** | -0.01 | -0.05, 0.03 | -0.004 | -0.05, 0.04 |
| **SHBG** | 0.004 | -0.03, 0.04 | 0.01 | -0.03, 0.05 |
|  | **Entorhinal cortex volume** | | | |
| **Total-T** | -0.03 | -0.08, 0.02 | -0.02 | -0.07, 0.03 |
| **Bio-T** | -0.01 | -0.06, 0.03 | -0.01 | -0.06, 0.04 |
| **SHBG** | -0.01 | -0.06, 0.03 | 0.002 | -0.05, 0.05 |
|  | **Pre-frontal cortex volume** | | | |
| **Total-T** | -0.81 | -1.82, 0.20 | -0.65 | -1.78, 0.49 |
| **Bio-T** | -0.27 | -1.31, 0.76 | -0.15 | -1.31, 1.01 |
| **SHBG** | -0.47 | -1.48, 0.54 | -0.22 | -1.32, 0.89 |
|  | **Medial-temporal volume** | | | |
| **Total-T** | -0.09 | -0.29, 0.12 | 0.02 | -0.18, 0.21 |
| **Bio-T** | 0.001 | -0.21, 0.21 | 0.03 | -0.16, 0.22 |
| **SHBG** | -0.13 | -0.33, 0.08 | -0.01 | -0.20, 0.17 |

Abbreviation: CI= confidence interval; T= testosterone; Bio-T= bioavailable testosterone; SHBG= sex-hormone binding globulin.

Levels of total-T, bio-T, and SHBG expressed in z-scores.

^a^Model 1 adjusted for age, education, race, and total intracranial volume.

^b^Model 2 adjusted for age, education, race, total intracranial volume, study center, body mass index, fasting insulin, hypercholesterolemia, smoking status, Apolipoprotein E e4 genotype, and history of vascular and cerebrovascular conditions, diabetes mellitus status, hypertension, and history of depressive symptoms.

**Supplementary Table 2. Sex-hormones levels and cognitive test scores: CARDIA men sub-sample with hormone and neuroimaging data**

|  | **Model 1^a^** | | **Model 2^b^** | | |
| --- | --- | --- | --- | --- | --- |
|  | Beta | 95% CI | Beta | 95% CI | |
|  | **Digit Symbol Substitution Test^c^** | | | | |
| **Total-T** | -0.03 | -0.14, 0.08 | -0.01 | -0.13, 0.11 | |
| **Bio-T** | 0.01 | -0.10, 0.12 | 0.02 | -0.10, 0.14 | |
| **SHBG** | -0.07 | -0.18, 0.04 | -0.06 | -0.18, 0.05 | |
|  | **Rey Auditory Verbal Learning Test^d^** | | | | |
| **Total-T** | -0.07 | -0.18, 0.04 | -0.09 | -0.22, 0.03 | |
| **Bio-T** | -0.01 | -0.13, 0.10 | -0.05 | -0.17, 0.08 | |
| **SHBG** | -0.06 | -0.17, 0.05 | -0.06 | -0.18, 0.06 | |
|  | **Stroop test^e^** | | | | |
| **Total-T** | -0.05 | -0.15, 0.06 | -0.04 | -0.16, 0.08 | |
| **Bio-T** | -0.12 | -0.22, -0.01 | -0.09 | -0.21, 0.03 | |
| **SHBG** | 0.06 | -0.05, 0.16 | 0.05 | -0.07, 0.16 |  |
|  | **Composite cognitive score^f^** | | | | |
| **Total-T** | -0.06 | -0.30, 0.19 | -0.06 | -0.33, 0.21 | |
| **Bio-T** | 0.12 | -0.13, 0.36 | 0.07 | -0.21, 0.34 | |
| **SHBG** | -0.20 | -0.45, 0.04 | -0.18 | -0.44, 0.08 | |

Abbreviation: CI= confidence interval; T= testosterone; Bio-T= bioavailable testosterone; SHBG= sex-hormone binding globulin.

Levels of total-T, bio-T, and SHBG and cognitive test scores expressed in z-scores.

^a^Model 1 adjusted for age, education, race, and total intracranial volume.

^b^Model 2 adjusted for age, education, race, total intracranial volume, study center, body mass index, fasting insulin, hypercholesterolemia, smoking status, Apolipoprotein E e4 genotype, and history of vascular and cerebrovascular conditions, diabetes mellitus status, hypertension, and history of depressive symptoms.

^c^Digit Symbol Substitution Test (DSST, psychomotor speed), higher scores indicate better cognitive performance.

^d^Rey Auditory Verbal Learning Test (RAVLT, verbal memory, expressed in a composite sum score of immediate and short-delay and long-delay recall); higher scores indicate better performance.

^e^Stroop test (executive function), higher scores indicate more interference in reaction time and worst cognitive performance.

^f^Composite score of cognitive function, generated by combining the z-scores of the three tests (RAVLT+DSST-STROOP); higher scores indicate better cognitive functioning.

**Supplementary Table 3.** **Sex-hormone levels at the three time-points of data collection (follow-up year 2, 7, and 10) and brain volumes of interest (at year 25)**

|  | **Year 2** | | | **Year 7** | | | **Year 10** | | |
| --- | --- | --- | --- | --- | --- | --- | --- | --- | --- |
|  | Beta^a^ | 95% CI | p | Beta^a^ | 95% CI | p | Beta^a^ | 95% CI | p |
| **Total white matter volume^b^** | | | | | | | | | |
| **Total-T** | -0.16 | -3.62, 3.30 | 0.929 | -0.08 | -3.69, 3.53 | 0.965 | 2.19 | -1.23, 5.62 | 0.209 |
| **Bio-T** | -0.56 | -4.04, 2.91 | 0.751 | -2.56 | -6.11, 0.99 | 0.157 | -1.78 | -5.40, 1.84 | 0.333 |
| **SHBG** | 1.01 | -2.46, 4.49 | 0.565 | **3.56** | **0.08, 7.03** | **0.045** | **4.04** | **0.68, 7.39** | **0.019** |
| **Total gray matter volume** | | | | | | | | | |
| **Total-T** | -0.74 | -3.68, 2.19 | 0.618 | -1.12 | -4.20, 1.95 | 0.472 | -2.98 | -5.95, -0.002 | 0.050 |
| **Bio-T** | 0.10 | -2.85, 3.05 | 0.948 | -0.04 | -3.08, 3.00 | 0.978 | 0.56 | -2.60, 3.71 | 0.729 |
| **SHBG** | -1.47 | -4.41, 1.48 | 0.327 | -2.14 | -5.11, 0.83 | 0.158 | -2.62 | -5.56, 0.32 | 0.081 |
| **Parietal gray matter volume** | | | | | | | | | |
| **Total-T** | -0.16 | -0.93, 0.61 | 0.685 | -0.03 | -0.82, 0.77 | 0.948 | -0.42 | -1.18, 0.35 | 0.281 |
| **Bio-T** | 0.45 | -0.33, 1.22 | 0.258 | -0.546 | -0.23, 1.33 | 0.169 | 0.53 | -0.28, 1.33 | 0.200 |
| **SHBG** | **-0.93** | **-1.69, -0.16** | **0.018** | **-0.77** | **-1.533, -0.01** | **0.048** | **-1.01** | **-1.76, -0.27** | **0.008** |

Abbreviation: CI= confidence interval; T= testosterone; Bio-T= bioavailable testosterone; SHBG= sex-hormone binding globulin.

^a^ Coefficients represent associations of individual sex-hormone levels at each time point (expressed in Z-scores) with brain volumes, estimated in adjusted Model 2 (including age, education, race, total intracranial volume, study center, body mass index, fasting insulin, hypercholesterolemia, smoking status, Apolipoprotein E e4 genotype, and history of vascular and cerebrovascular conditions, diabetes mellitus status, hypertension, and history of depressive symptoms).

**^b^** Same pattern is observed with lobar volumes; SHBG marginally associated with frontal white matter volume (p=0.063 and p=0.055 for Year 7 and Year 10 SHBG levels respectively) and with temporal white matter volume (p=0.084 and p=0.017 for Year 7 and Year 10 SHBG levels respectively).

**Supplementary Table 4. Sample’s characteristics and their associations with sex-hormones levels: CARDIA men sub-sample with hormone and neuroimaging data**

|  | **Total-T^a^** | | **Bio-T^a^** | | **SHBG^a^** | |
| --- | --- | --- | --- | --- | --- | --- |
|  | Beta^b^ | 95% CI | Beta^b^ | 95% CI | Beta^b^ | 95% CI |
| Age | -0.03 | -0.07, 0.003 | -0.05** | -0.09, -0.02 | 0.03 | -0.01, 0.06 |
| Race *(black)* | 0.10 | -0.15, 0.35 | 0.36** | 0.11, 0.61 | -0.15 | -0.40, 0.11 |
| Education *(high school or less)* | -0.06 | -0.35, 0.22 | 0.01 | -0.28, 0.29 | 0.06 | -0.35, 0.22 |
| Smoking status |  |  |  |  |  |  |
| Never-smoker | Ref. |  | Ref. |  | Ref. |  |
| Ex-smoker | 0.10 | -0.21, 0.41 | 0.01 | -0.30, 0.32 | 0.02 | -0.32, 0.29 |
| Smoker | 0.12 | -0.22, 0.47 | 0.29 | -0.05, 0.64 | 0.06 | -0.41, 0.28 |
| APOEe4 genotype *(e4 carrier)* | 0.09 | -0.17, 0.35 | -0.08 | -0.35, 0.19 | 0.08 | -0.35, 0.19 |
| History of diabetes mellitus status | -0.48** | -0.87, -0.08 | -0.30 | -0.70, 0.10 | 0.37 | -0.77, 0.02 |
| History of cardio-vascular events | -0.02 | -0.40, 0.36 | -0.31 | -0.69, 0.08 | 0.10 | -0.28, 0.49 |
| History of hypertension | -0.32** | -0.56, -0.07 | -0.26** | -0.51, -0.01 | 0.19 | -0.44, 0.06 |
| History of depressive symptoms | 0.03 | -0.22, 0.28 | -0.01 | -0.25, 0.24 | 0.03 | -0.28, 0.22 |
| BMI | -0.07** | -0.09, 0.04 | -0.02 | -0.05, 0.003 | 0.07** | -0.09, -0.04 |
| Fasting insulin *(uU/ml)* | -0.02** | -0.028, -0.003 | -0.01 | -0.02, 0.001 | 0.02** | -0.03, -0.003 |
| Hypercholesterolemia | -0.21 | -0.501, 0.08 | -0.24 | -0.53, 0.05 | 0.13 | -0.42, 0.16 |
| Composite cognitive score | -0.01 | -0.06, 0.04 | 0.003 | -0.05, 0.06 | 0.03 | -0.08, 0.03 |

**p<0.05

Abbreviation: APOE= apolipoprotein; BMI= body mass index; CI= confidence interval; T= testosterone; Bio-T= bioavailable testosterone; SHBG= sex-hormone binding globulin.

^a^Hormone measures expressed in z-scores.

^b^Unadjusted betas computed using univariate linear regressions.

**Supplementary Table 5. Sample’s characteristics and their associations with brain volumes: CARDIA men sub-sample with hormone and neuroimaging data**

|  | **Total gray matter volume** | | **Total white matter volume** | |
| --- | --- | --- | --- | --- |
|  | Beta^a^ | 95% CI | Beta^a^ | 95% CI |
| Age | -0.99 | -2.78, 0.79 | -0.49 | -2.42, 1.43 |
| Race *(black)* | -42.32** | -54.48, -30.15 | -26.50** | -40.34, -12.65 |
| Education *(high school or less)* | -10.47 | -25.35, 4.40 | -5.72 | -21.78, 10.34 |
| Smoking status |  |  |  |  |
| Never-smoker | Ref. |  | Ref. |  |
| Ex-smoker | 1.74 | -14.03, 17.50 | 2.24 | -14.92, 19.39 |
| Smoker | -25.58** | -43.26, -7.89 | -12.96 | -32.20, 6.29 |
| APOEe4 genotype *(e4 carrier)* | 1.29 | -12.70, 15.27 | 8.96 | -5.84, 23.76 |
| History of diabetes mellitus status | -23.36** | -44.07, -2.64 | -17.12 | -39.54, 5.30 |
| History of cardio-vascular events | 1.51 | -18.45, 21.46 | 11.71 | -9.74, 33.15 |
| History of hypertension | -23.28** | -35.92, -10.64 | -19.42** | -33.16, -5.67 |
| History of depressive symptoms | -18.84** | -31.61, -6.07 | -20.27** | -34.02, -6.52 |
| BMI | -0.36 | -1.74, 1.20 | -0.66 | -2.14, 0.83 |
| Fasting insulin *(uU/ml)* | -0.22 | -0.88, 0.45 | -0.52 | -1.23, 0.20 |
| Hypercholesterolemia | 3.18 | -12.01, 18.37 | -2.71 | -19.06, 13.65 |
| Cognitive score | 7.39** | 4.72, 10.06 | 5.44** | 2.48, 8.40 |

**p<0.05

Abbreviation: APOE= apolipoprotein; BMI= body mass index; CI= confidence interval; T= testosterone; Bio-T= bioavailable testosterone; SHBG= sex-hormone binding globulin.

^a^Unadjusted betas computed using univariate linear regressions; positive betas indicate association with larger volumes and negative betas with smaller volumes.
